# A Fusion Algorithm for Pedestrian Anomaly Detection and Tracking on Urban Roads Based on Multi-Module Collaboration and Cross-Frame Matching Optimization

**DOI:** 10.3390/s26020400

**Published:** 2026-01-08

**Authors:** Wei Zhao, Xin Gong, Lanlan Li, Luoyang Zuo

**Affiliations:** School of Vehicle and Traffic Engineering, Henan University of Science and Technology, Luoyang 471003, China; 230320030344@stu.haust.edu.cn (X.G.); 230320030388@stu.haust.edu.cn (L.L.); 240320030337@stu.haust.edu.cn (L.Z.)

**Keywords:** abnormal behavior detection, improved YOLOv8, BoT-SORT-ReID, detection-tracking integrated model

## Abstract

Amid rapid advancements in artificial intelligence, the detection of abnormal human behaviors in complex traffic environments has garnered significant attention. However, detection errors frequently occur due to interference from complex backgrounds, small targets, and other factors. Therefore, this paper proposes a research methodology that integrates the anomaly detection YOLO-SGCF algorithm with the tracking BoT-SORT-ReID algorithm. The detection module uses YOLOv8 as the baseline model, incorporating Swin Transformer to enhance global feature modeling capabilities in complex scenes. CBAM and CA attention are embedded into the Neck and backbone, respectively: CBAM enables dual-dimensional channel-spatial weighting, while CA precisely captures object location features by encoding coordinate information. The Neck layer incorporates GSConv convolutional modules to reduce computational load while expanding feature receptive fields. The loss function is replaced with Focal-EIoU to address sample imbalance issues and precisely optimize bounding box regression. For tracking, to enhance long-term tracking stability, ReID feature distances are incorporated during the BoT-SORT data association phase. This integrates behavioral category information from YOLO-SGCF, enabling the identification and tracking of abnormal pedestrian behaviors in complex environments. Evaluations on our self-built dataset (covering four abnormal behaviors: Climb, Fall, Fight, Phone) show mAP@50%, precision, and recall reaching 92.2%, 90.75%, and 86.57% respectively—improvements of 3.4%, 4.4%, and 6% over the original model—while maintaining an inference speed of 328.49 FPS. Additionally, generalization testing on the UCSD Ped1 dataset (covering six abnormal behaviors: Biker, Skater, Car, Wheelchair, Lawn, Runner) yielded an mAP score of 92.7%, representing a 1.5% improvement over the original model and outperforming existing mainstream models. Furthermore, the tracking algorithm achieved an MOTA of 90.8% and an MOTP of 92.6%, with a 47.6% reduction in IDS, demonstrating superior tracking performance compared to existing mainstream algorithms.

## 1. Introduction

Detecting and identifying abnormal pedestrian behavior in complex traffic environments is a task in computer vision that combines both technical depth and practical significance. Anomalous behavior refers to actions, phenomena, or objects that deviate from expected patterns within a specific scenario [1]. Characterized by unpredictability, non-periodicity, short duration, and high suddenness, such behaviors prove difficult to capture precisely through fixed rules. The primary task of anomaly behavior detection is to identify known categories of abnormal behavior based on images and discover novel patterns of unknown anomalous categories [2].

In recent years, pedestrian anomaly detection based on deep learning has emerged as a prominent research focus in computer vision, playing a vital role across numerous practical applications. For instance, in autonomous driving, this technology effectively identifies sudden pedestrian incidents on roadways and performs real-time dynamic analysis, assisting drivers in safe operation and significantly reducing traffic accidents. In traffic management, it can swiftly identify abnormal behaviors, such as drivers using phones or driving while fatigued [3], enabling timely intervention and enhanced warnings to reduce distracted driving risks and prevent accidents effectively. The ultimate goal of abnormal human behavior research is to liberate human vision, replace traditional surveillance systems with their low recognition rates and high false-negative rates, and accomplish real-time automated early warning tasks. Therefore, this research holds significant practical importance.

### 1.1. Object Detection Algorithm

The development of object detection algorithms can be classified into two phases: traditional algorithms and deep learning algorithms [4].

Traditional algorithms primarily detect abnormal behavior through “manually designed features + classical machine learning classifiers.” Based on technical approaches, they can be categorized into three major types: sliding window-based detection algorithms, region proposal-based detection algorithms, and statistical learning-based detection algorithms. Manual feature extraction relies on gradient histograms (HOG) [5], motion flow, optical flow features [6], sparse coding [7], trajectory features, scale-invariant feature transform (SIFT), and other low-level visual features. The extracted feature vectors are then fed into classical machine learning classifiers (such as SVM, AdaBoost, etc.) for classification and localization. Dalal [8] pioneered the integration of HOG features with SVM for pedestrian detection, establishing the “handcrafted features + SVM” paradigm for this task. Wang et al. [9] combined multiple features of abnormal behavior to propose a multi-feature fusion approach for detecting such behaviors, overcoming the limitations of single features and achieving greater robustness. Shih-Chung et al. [10] innovatively combined unsupervised learning with SVM methods to construct a two-stage detection framework for distinguishing between normal and abnormal human behaviors, but they did not thoroughly explore the impact of complex scenes on unsupervised feature extraction. In summary, traditional anomaly detection algorithms exhibit distinct strengths and weaknesses: their advantages lie in low computational demands and minimal hardware requirements, particularly advantageous in scenarios with limited sample data. Their disadvantages include manually extracted features that are often scenario-specific, resulting in limitations, singularity, weak robustness, and poor generalization capabilities.

With technological advancements, deep learning techniques have demonstrated increasingly impressive performance in addressing visual challenges. Leveraging their exceptional detection capabilities, they have emerged as the mainstream approach for object detection research in recent years. Common algorithms include two types:

(1) Two-stage detection algorithms, such as Region-based Convolutional Neural Networks (R-CNN) [11], Fast R-CNN [12], Faster R-CNN [13], and Mask R-CNN [14]. This type of algorithm operates in two stages: first, generating candidate regions, then predicting and precisely identifying the object locations within these regions to achieve higher detection accuracy. However, it suffers from slow detection speeds, low efficiency, and poor real-time performance. Mehmood [15] integrated 3D convolutions into both spatial and temporal streams to construct a dual-stream 3D CNN architecture, significantly improving anomaly detection accuracy in non-crowded scenarios. Pathak et al. [16] designed a CNN-LSTM autoencoder, leveraging CNN to extract spatial features and LSTM to model temporal dependencies, enabling end-to-end spatio-temporal feature learning. PS et al. [17] combined CNN-LSTM with the Chimpanzee Optimization Algorithm (ChoA), incorporating bidirectional filtering preprocessing and multimodal feature fusion to enhance robustness in complex scenarios.
(2) One-stage detection algorithms, such as Single Shot MultiBox Detection (SSD) [18], YOLO Algorithm Series [19], RetinaNet, DETR, et al. These algorithms directly predict object categories and bounding boxes on the input image instead of generating extra candidate regions, significantly reducing computational load and enabling more real-time and efficient detection. These approaches have now become mainstream methods. Ji et al. [20] proposed the T-TINY-YOLO network model, employing a deep separable convolution for feature reconstruction, eliminating redundant detection heads, and incorporating lightweight LSTM modules in series to achieve end-to-end classification of human abnormal behaviors. Xu et al. [21] replaced the original VGG16 backbone network of SSD with MobileNetV2, incorporated a channel attention module for multi-scale feature fusion to achieve a lightweight design, and implemented dynamic threshold adjustment, making it suitable for scenarios requiring rapid response. Fang et al. [22] optimized the backbone network and feature fusion architecture of YOLOv3, redesigning bounding box parameters to deliver a practical solution for scenarios with stringent requirements. Rong et al. [23] adopted DETR as the baseline model, introduced deformable attention encoding, and employed an improved EfficientNet backbone that incorporates the Channel-Batch Attention Module (CBAM) to enhance key feature extraction. Combining Smooth-L1 and GIoU as loss functions during training accelerates model convergence and significantly boosts detection accuracy.

### 1.2. Object Tracking Algorithm

Multi-target tracking tasks typically adopt the Tracking-by-Detection (TBD) paradigm [24], where target detection accuracy directly determines tracking performance. It is crucial to address occlusion issues between moving targets and their backgrounds, as well as among multiple moving targets. Parate et al. [25] proposed a lightweight, modular framework for pedestrian anomaly detection tailored for IoT edge devices. Employing feature encoding and trajectory association techniques, it leverages two complementary metrics to address partial occlusion, pose deformation, and complex scenes, achieving satisfactory detection performance while maintaining real-time capability. Zhao et al. [26] proposed a lightweight tracking framework based on an improved DeepSORT, employing ShuffleNetV2-1.0 as the ReID base network. By implementing Channel Shuffle and pointwise batch convolutions, we significantly reduced both the number of parameters and computational complexity. Feng et al. [27] proposed a feature embedding enhancement scheme centered on Graph Attention Networks (GAT), constructing the SCGTracker framework. They designed a multimodal association mechanism and introduced temporal constraints to enhance trajectory association stability. Roka et al. [28] constructed a three-feature association (Appearance (ReID) + Motion (trajectory trend) + Camera Topology (location mapping)) to achieve cross-camera pedestrian tracking continuation. They replaced the DeepSORT backbone network with MobileNetV3 and performed channel pruning, enhancing tracking efficiency through multi-camera multi-stream tracking coordination.

Despite progress in existing pedestrian anomaly detection and tracking research, three critical gaps remain: First, most detection algorithms are based on single, interference-free ideal scenarios, making them difficult to adapt to complex real-world environments. Second, detection and tracking modules are often designed independently, with insufficient exploration of their technical interdependencies. This leads to poor coordination, low efficiency, and unstable tracking during integrated applications. Third, existing algorithms exhibit significant variations in generalization performance across custom scenarios and public datasets, making them ill-suited for diverse urban road environments. To address these challenges, this paper proposes a multi-module collaborative fusion algorithm. It constructs a joint recognition framework combining an improved YOLOv8n as the detector with the Bot-SORT-ReID tracking module. This framework detects and tracks four high-frequency abnormal behaviors prone to causing safety incidents in urban road scenarios: Fall, Fight, Climb, Phone. Through structural optimization of the detection module, feature fusion in the tracking module, and cross-module information coordination, the approach achieves simultaneous improvements in detection accuracy, tracking stability, and generalization capability. This advances the practical application of integrated anomaly detection-tracking technology in complex traffic scenarios.

## 2. Materials and Methods

By enhancing the multi-scale feature fusion capabilities through improvements to the YOLOv8n network architecture and introducing an attention mechanism to boost sensitivity to abnormal behavior features, detection accuracy and recall rates in complex backgrounds are effectively elevated. The integration of a deep fusion mechanism for semantic features and motion trajectories within the Bot-SORT-ReID framework enhances identity consistency under challenges such as occlusion and deformation, significantly improving the stability and continuity of long-term tracking. A bidirectional feedback pathway between detection and tracking has been designed to dynamically calibrate and complementarily optimize detection results with tracking trajectories. This prevents error accumulation and performance degradation caused by isolated modules, thereby enhancing the system’s overall responsiveness and collaborative capabilities.

### 2.1. YOLOv8 Object Detection

**Figure 1:**
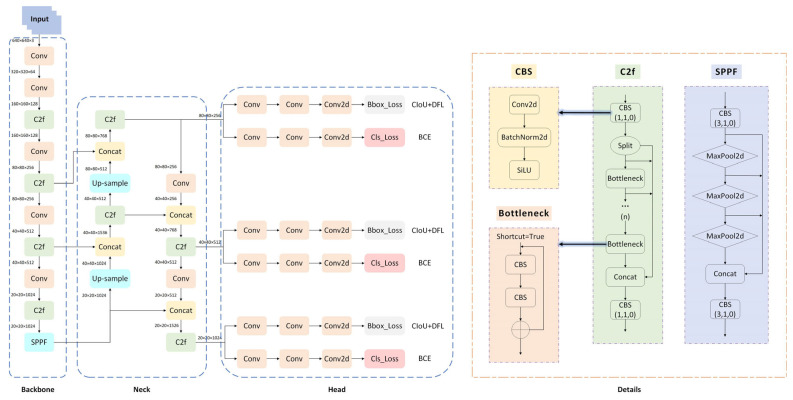
YOLOv8 network architecture and key modules diagram. The blue boxes represent the Backbone, Neck, and Head sections of the model, while the orange boxes show the detailed representations of specific modules.

YOLOv8 [29], released by Ultralytics in 2023, offers five different-scale versions—N, S, M, L, X—based on network depth, width, and maximum channel count, enabling adaptation to various deployment platforms and scenarios. Among these, YOLOv8n features the fewest parameters and minimal floating-point operations, delivering high efficiency ideal for real-time deployment while maintaining strong accuracy. Consequently, this paper adopts YOLOv8n as the baseline model, whose network architecture and key components are illustrated in Figure 1.

YOLOv8 abandons traditional residual structures, integrating C3 modules with ELAN concepts to introduce C2F modules. These modules process and fuse input features through segmentation, enhancing gradient information flow and feature extraction capabilities while maintaining high computational efficiency. The Neck section adopts the PAN-FPN architecture to integrate multi-scale features and introduces SPPF modules to expand receptive fields through fast serial pooling. The Head employs the Decoupled-Head architecture to separate classification and regression tasks. For the loss function, the regression branch innovatively combines DFL with CIOU loss, jointly improving target localization accuracy.

Despite its outstanding performance, YOLOv8 still faces numerous challenges in pedestrian detection within complex scenarios. These include heightened demands for lightweight and real-time capabilities in resource-constrained environments, as well as the need to improve detection accuracy and stability in crowded areas and scenes with complex lighting conditions. Therefore, this paper proposes the YOLO-SGCF algorithm as an enhancement to the YOLOv8 model, with the following specific optimizations:

- The Swin Transformer module is introduced into the backbone, dynamically balancing detail and semantic information through self-attention mechanisms. This replaces static local convolutional operations with dynamic global attention mechanisms, enhancing the modeling capability of global features.
- Embed CBAM and CA attention modules in the Neck and Backbone, respectively. CA enhances feature positional sensitivity and scene relevance, delivering high-quality, positionally accurate raw features; CBAM strengthens cross-scale feature selection and focus, outputting refined features better suited for prediction.
- The Neck section introduces GSConv modules to replace conventional convolutions. Group Convolution reduces computational overhead for multi-scale fusion, while Channel Shuffle prevents feature fragmentation. This enables the Neck to efficiently process multi-scale features while outputting richer, more strongly correlated fused features.
- The loss function is replaced with Focal-EIoU. By optimizing width-height error calculations and focusing on challenging samples, this enhances bounding box regression accuracy.

**Figure 2:**
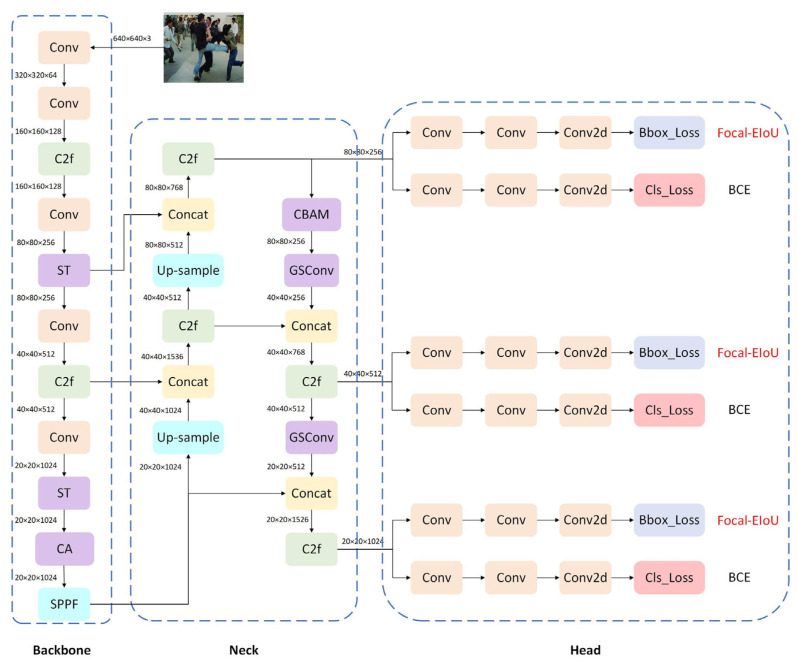
YOLO-SGCF network architecture diagram.

The YOLO-SGCF model framework is illustrated in Figure 2.

#### 2.1.1. Swin Transformer

Swin Transformer [30] is a Transformer-based visual backbone network proposed in 2021. Compared to traditional convolutional neural networks (CNNs), Swin Transformer can more flexibly model global dependencies and demonstrates outstanding performance across multiple visual tasks.

**Figure 3:**
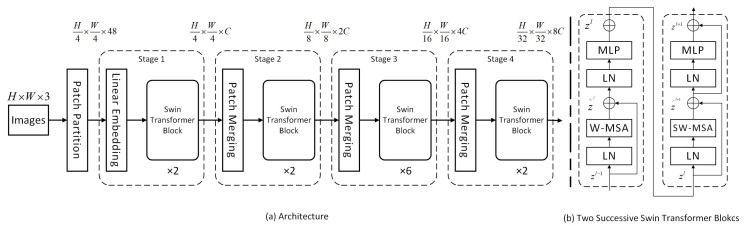
Swin Transformer network architecture diagram. (**a**) depicts the complete network architecture flow of the Swin Transformer; (**b**) focuses on the internal structure of the Swin Transformer Block, illustrating how two consecutive Swin Transformer modules operate. The “⊕” symbol represents the addition operation in residual connections, where input features are added element-wise to features processed by the module. This preserves original feature information and mitigates the vanishing gradient problem in deep networks. The z represents the feature tensor, and the superscript l denotes the module’s layer level.

In real-world scenarios, pedestrians vary in distance from the camera, leading to significant scale differences in their representations within images. Conventional CNNs may lose fine-grained details of small-scale pedestrians or fail to fully capture features of large-scale pedestrians when processing objects at different scales. The Swin Transformer’s multi-scale feature fusion capability effectively addresses this scale variation issue, enhancing the detection of abnormal behaviors across pedestrians of varying sizes. The structural diagram is shown in Figure 3.

The entire model adopts a hierarchical design comprising four stages. The Patch Partition layer primarily handles preprocessing of the input image, segmenting the original image into non-overlapping M×M patches. These image blocks are then unfolded into one-dimensional vectors and mapped to a high-dimensional feature space via a linear embedding operation, while simultaneously incorporating position embeddings. The Patch Merging layer implements downsampling to construct the hierarchical feature representation of the Swin Transformer. It concatenates the features of each set of 2×2 adjacent patches and applies a linear layer to the resulting high-dimensional features. Subsequently, a Swin Transformer Block performs feature transformation, maintaining a resolution of H/8×W/8. The patch merging and feature transformation in the first block constitute “Stage 2.” This process repeats twice as “Stage 3” and “Stage 4.”

The Swin Transformer Block typically appears as a two-stage serial structure. In the first stage, Window-based Multi-headed Self-Attention (W-MSA) is employed to divide the feature map into multiple non-overlapping windows. Self-attention is computed only within each window, significantly reducing computational complexity. The W-MSA module first divides the feature map into multiple windows. Assuming each window has dimensions M in height and width, this yields a total of h/M×w/M windows. A multi-headed attention module is then applied within each window. The computational complexity for processing a feature map with height h, width w, and C channels is as follows:

(1) $$4hwC^{2}+2{\left(hw\right)}^{2}C=4{\left(MC\right)}^{2}+2{\left(M\right)}^{4}C$$

Since there are h/M×w/M windows, the calculation formula is as follows:

(2) $$\frac{h}{M}\times \frac{w}{M}\times \left(4{\left(MC\right)}^{2}+2{\left(MC\right)}^{4}C\right)=4hwC^{2}+2M^{2}hwC$$

The second stage employs Shifted Window Multi-Head Self-Attention (SW-MSA). By shifting the window boundaries, it overcomes the limitations of the W-MSA module, enabling cross-window information exchange to effectively capture global feature dependencies. Each attention module is followed by a standard structure comprising an MLP, a residual connection, and layer normalization. The MLP further extracts high-level features through nonlinear transformations; the residual connection directly adds the module’s input and output, effectively mitigating gradient vanishing and aiding deep network training; layer normalization stabilizes the training process and accelerates convergence by normalizing the feature distribution.

Overall, the Swin Transformer balances modeling capabilities with the efficiency demands of visual tasks through Window Attention and Shifted Window Attention. It preserves global context modeling capabilities while significantly enhancing computational efficiency for visual tasks.

#### 2.1.2. GhostConv

GhostConv [31] is a lightweight convolution operation, designed to significantly reduce the computational load and parameter count of convolutional neural networks without substantially compromising model performance.

**Figure 4:**
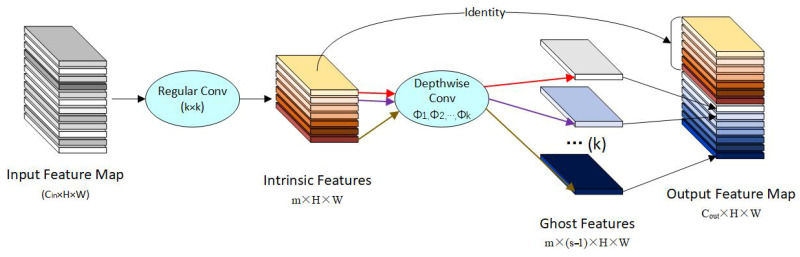
Block diagram of GhostConv.

GhostConv achieves model lightweighting by decomposing traditional convolution steps. It first generates partial intrinsic feature maps using a small number of convolutional kernels, then applies low-cost linear transformations to these maps to produce additional ghost feature maps, and finally concatenates the two sets. This approach significantly reduces computational cost and parameter count while maintaining performance, making it more suitable for resource-constrained deployment environments. The structural diagram of GhostConv is shown in Figure 4, with the specific workflow as follows:

(1) Input Feature Map: Input the original feature map with dimensions $C_{in}\times H\times W$.
(2) Regular Convolution: Performs convolution operations on the input feature map using a small number of convolution kernels (typically k × k convolution). This step aims to extract key information from the input feature map, generating intrinsic features with dimensions m×H×W. This computation is relatively light but captures the primary characteristics of the feature map.
(3) Depthwise Convolution: Apply linear transformations to the intrinsic features obtained in the first step. Using different convolution kernels $\phi_{1\;},\phi_{2\;},\ldots ,\phi_{k}$, generate ghost features with dimensions m×(s−1)×H×W (where s is a scaling factor controlling the number of generated ghost features). Linear transformation typically employs simpler methods like depthwise convolution, which has low computational cost and can generate large numbers of feature maps without excessive computational overhead.
(4) Identity: Directly preserves the intrinsic features (without additional computation) as the base features.
(5) Feature Map Concatenation: Concatenate the intrinsic features with the generated ghost features along the channel dimension to obtain the Output Feature Map. The final output channel count is $m+m\times \left(s-1\right)=m\times s=C_{out}$, with an output size of $C_{out}\times H\times W$. This approach preserves the critical information from the original feature map while enhancing feature diversity through the ghost feature map.

The Neck component primarily handles the fusion of feature maps at different scales. It performs operations such as upsampling, downsampling, and concatenation on the multi-scale features output by the Backbone, thereby further extracting and adjusting the features. During this process, GhostConv can leverage its lightweight nature to reduce computational resource consumption and accelerate model inference speed. Despite minimizing computational load and parameter count, GhostConv still generates rich feature maps, ensuring effective fusion of multi-scale features and enhancing the model’s detection accuracy for objects of varying sizes.

#### 2.1.3. Attention

1. CA

CA (Coordinate Attention Mechanism) [32] is an attention mechanism designed for CNN. While traditional attention mechanisms typically calculate attention weights based on relative or absolute position information, CA introduces coordinate position information as the basis for attention computation. It primarily addresses how to better capture spatial information and inter-channel dependencies within the network.

**Figure 5:**
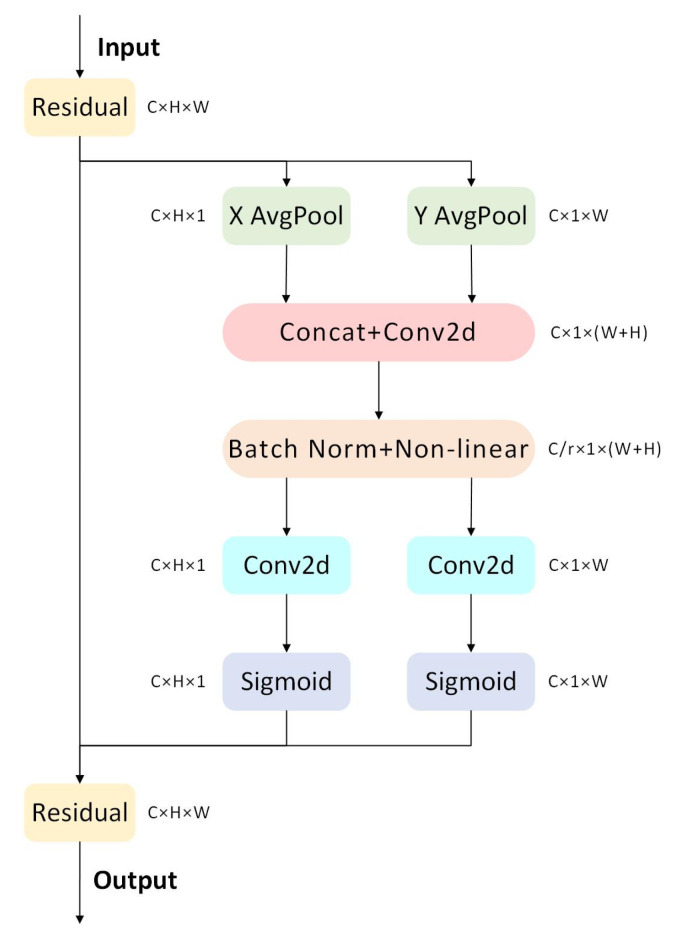
CA architecture diagram.

The core concept of CA is to take the coordinates of samples as input and compute attention weights based on the relative distances between different positions in space. These attention weights can be used to weight different positions within the input feature map, thereby obtaining more locally specific feature representations. The specific process is illustrated in Figure 5.

The input feature map is $X\in R^{C\times H\times W}$. It first passes through a residual module, which preserves the fundamental information of the original features while providing input for subsequent attention calculations. Horizontal (X AvgPool) and vertical (Y AvgPool) average pooling are then performed separately. Taking X AvgPool as an example, global average pooling is applied along the horizontal dimension (width W) of the input feature map, yielding the horizontal feature vector $X_{pool}\in R^{C\times H\times 1}$. The formula is:

(3) $$X_{pool}\left(c,h\right)=\sum_{w=1}^{W}X(c,h,w)$$

where c∈{1,2,…,C}, w∈{1,2,…,W}.

The horizontally pooled $X_{pool}$ and vertically pooled $Y_{pool}$ are concatenated along the channel dimension (Concat), yielding the concatenated feature. Then, a 1 × 1 convolution (Conv2d) operation is performed on this feature to fuse horizontal and vertical information. Batch normalization (Batch Norm) is then applied to the convolved feature to accelerate training and enhance stability. Then, it is passed through a non-linear activation function, such as ReLU, to obtain the activated feature $F_{act}\in R^{C/r\times 1\times (W+H)}$ (where r is the reduction rate, used to reduce computational load). $F_{act}$ is then separated into a horizontal feature $F_{x}$ and vertical feature $F_{y}$, a 1 × 1 convolution operation is performed, and the Sigmoid activation function is applied to each feature to obtain the horizontal attention weight $M_{x}\in R^{C\times H\times 1}$ and vertical attention weight $M_{y}\in R^{C\times 1\times W}$. Each weight is multiplied element-wise with the residual feature of the original input; the results are summed, and finally passed through the residual module to obtain the output feature $O\in R^{C\times H\times W}$. The formula is as follows:

(4) $$O=Residual(X{⨀M}_{x}⨀M_{y})$$

Unlike previous approaches that convert 2D global pooling into a single feature vector, CA decomposes channel attention into 1D feature encoding with bidirectional aggregation. This approach captures long-range dependencies and precise positional information along two distinct directions, generating complementary feature maps to enhance target representations. This method avoids the loss of positional information inherent in 2D global pooling, thereby more effectively improving network performance.

In response to this characteristic, this paper embeds the CA into the backbone of YOLOv8. This allows the network to focus more on target-related regions and channel features within images, enabling it to capture key features of abnormal behavior more accurately while suppressing interference from irrelevant background information. Simultaneously, it enables the subsequent Neck and Head components to perform object bounding box regression and category prediction based on more precise features. This significantly improves the accuracy of target location detection, particularly for multi-object detection in complex scenes.

2. CBAM

**Figure 6:**
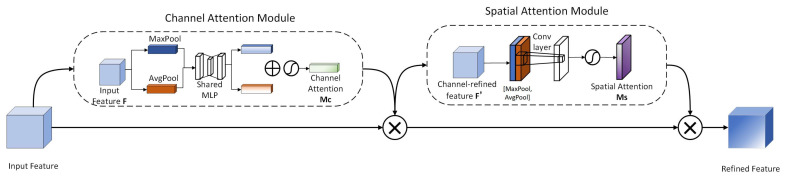
Structure of the CBAM attention.

CBAM (Convolutional Block Attention Module) [33] is a representative lightweight attention mechanism. Its core philosophy lies in integrating two major branches: Channel Attention and Spatial Attention. The module’s significant advantage lies in its ability to effectively enhance a convolutional neural network’s capability to capture and utilize key features, thereby improving the model’s feature extraction performance, with minimal increase in overall computational complexity and parameter count. The specific structure and workflow are illustrated in Figure 6.

The primary objective of CAM is to explicitly model dependencies between channels, generating a channel attention map. First, the input feature map $F\in R^{C\times H\times W}$ undergoes both Global Average Pooling (GAP) and Global Max Pooling (GMP) operations. This reduces the spatial dimension of the input features to 1, generating two distinct channel descriptors. The calculation formulas are as follows:

(5) $$F_{avg}^{c}=\frac{1}{H\times W}\sum_{i=1}^{H}F^{c}(i,j)$$

(6) $$F_{max}^{c}={max}_{i=1}^{H}{max}_{j=1}^{W}F^{c}(i,j)$$

where $F^{c}(i,j)$ denotes the value at coordinates (i,j) in the cth channel of the feature map F.

Then, the two channel descriptors are input into a shared multilayer perceptron (MLP) for feature transformation, which consists of a hidden layer and an output layer, with the hidden layer containing C/r neurons, where r is the dimension reduction factor. Let the weight matrices of the multilayer perceptron be $W_{1}\in R^{C/r\times C}$ and $W_{2}\in R^{C\times C/r}$, with biases $b_{1}\in R^{C/r}$ and $b_{2}\in R^{C}$. The result of global average pooling is as follows:

(7) $$M_{avg}^{c}=\sigma (W_{2}\left(\delta \left(W_{1}F_{avg}^{c}+b_{1}\right)\right)+b_{2})$$

Global max pooling is:

(8) $$M_{max}^{c}=\sigma (W_{2}\left(\delta \left(W_{1}F_{max}^{c}+b_{1}\right)\right)+b_{2})$$

where σ represents the sigmoid activation function, and δ represents the ReLU activation function.

The two weights are added to obtain the channel attention weight $M_{c}\in R^{C\times 1\times 1}$.

The primary objective of SAM is to explicitly model spatial dependencies between locations, generating a spatial attention map. First, the input feature map $F\in R^{C\times H\times W}$ undergoes max pooling and average pooling along the channel dimension, yielding two H×W feature maps.

These two feature maps are concatenated along the channel dimension to form a 2×H×W feature map. This is then processed through a 7 × 7 convolutional layer (with 1 kernel, stride 1, and padding 3) to learn spatial correlations. The values of the spatial attention map are then normalized to the range [0, 1] via a Sigmoid activation function, yielding the spatial attention weights $M_{S}\in R^{1\times H\times W}$. The calculation formula is:

(9) $$M_{S}=\sigma (f^{7\times 7}([F_{avg}^{S};F_{max}^{S}]))$$

where $f^{7\times 7}$ denotes a 7 × 7 convolution operation, while [;] represents a concatenation operation along the channel dimension.

Ultimately, the channel attention weights $M_{C}$ and spatial attention weights $M_{S}$ are multiplied element-wise with F in sequence, yielding the attention-adjusted feature map $F^{\prime }$.

The combination of channel and spatial attention mechanisms has proven effective in complex traffic detection tasks. Tan et al. [34] integrated both attention mechanisms into a deep differential segmentation network for rail transit foreign object detection, successfully mitigating interference from airflow disturbances and lighting variations, thereby validating the complementary advantages of dual-dimensional attention. In pedestrian anomaly detection scenarios, abnormal movements often involve distinctive postural changes across body parts and interactions with the surrounding environment, necessitating precise capture of key information from multi-scale features. CBAM employs channel attention to enable the network to focus on feature channels crucial to target behaviors—such as those related to human contours, dynamic postural changes, and anomalous actions. Spatial attention highlights the spatial region containing the target within the image, reducing background noise interference for more accurate target recognition and localization. Furthermore, during multi-scale feature fusion, CBAM performs separate attention calculations and adjustments for feature maps at different scales. This enables more rational weight allocation during fusion, enhancing valuable features at various scales that capture details of pedestrian abnormal actions. Consequently, the effectiveness of multi-scale feature fusion is significantly improved.

#### 2.1.4. Focal-EIoU Loss

The Intersection over Union (IoU) loss function measures the degree of overlap between predicted and ground-truth bounding boxes, thereby evaluating the accuracy of predicted boxes. YOLOv8 employs the CIoU loss function. For low-quality images, unclear edge information of objects may cause significant positional errors between predicted and ground-truth boxes, leading to poor detection performance. EIoU improves upon CIoU by separating the aspect ratio into width and height components, calculating the difference values for each dimension. The width-height loss directly minimizes the difference between the width and height of the predicted box and the ground truth box, accelerating convergence. However, it lacks a dedicated mechanism for handling sample imbalance, leading to simple samples dominating training while difficult samples receive insufficient optimization. Focal Loss [35] addresses this by reducing the loss weight of easy samples, enabling the model to focus on hard samples and enhance overall detection robustness. Therefore, when selecting loss functions, we introduce a dynamic weighting mechanism building upon EIoU that assigns higher weights to samples with low IoU. This optimizes the sample imbalance issue in bounding box regression, compelling the model to focus on challenging samples.

Given two boxes M and N with areas $A_{M}$ and $A_{N}$, respectively, their IoU is defined as:

(10) $$IoU\left(M,N\right)=\frac{M\cap N}{M\cup N}$$

where M∩N denotes the intersection region between region M and region N, denoted as $A_{MN}$, and M∪N denotes the union region between region M and region N. Then we have:

(11) $$IoU\left(M,N\right)=\frac{M\cap N}{M\cup N}=\frac{A_{MN}}{A_{M}+A_{N}-A_{MN}}$$

A higher IoU value indicates greater prediction accuracy. Conversely, a lower IoU indicates poorer model performance.

Compared to the CIoU penalty term, the core optimization of the EIoU penalty lies in separating the aspect ratio influence factor to calculate the length and width of the target box and prediction box independently. This loss function comprises three components: the overlap loss $L_{IoU}$, the center distance loss $L_{dis}$, and width-height loss $L_{asp}$. The first two components align with CIoU. $L_{asp}$ accelerates convergence by directly reducing the gap between the target bounding box and the predicted bounding box in width and height. The specific penalty term formula is as follows:

(12) $$L_{EIoU}=L_{IoU}+L_{dis}+L_{asp}=1-IoU+\frac{p^{2}\left(b,b^{gt}\right)}{{c_{w}}^{2}+{c_{h}}^{2}}+\frac{p^{2}\left(w,w^{gt}\right)}{{c_{w}}^{2}}+\frac{p^{2}\left(h,h^{gt}\right)}{{c_{h}}^{2}}$$

where $c_{w}$ and $c_{h}$ represent the width and height of the minimum bounding box covering both boxes.

By integrating EIoU Loss and Focal L1 loss, the Focal-EIoU loss formula is derived:

(13) $$L_{Focal-EIoU}={IoU}^{\gamma }L_{EIoU}$$

where γ is a hyperparameter that controls the degree of outlier suppression, regulating the curvature of the curve.

### 2.2. BoT-SORT-ReID Tracking Module

BOT-SORT (Box and Occlusion-aware Tracking with Transformer) is an algorithm for multi-object tracking (MOT) designed to enhance tracking robustness and accuracy. It integrates object detection with association strategies, achieving efficient tracking by optimizing the sorting and assignment processes within the tracking framework. Most SORT (Simple Online and Realtime Tracking) algorithms typically employ Kalman filtering based on the uniform motion model assumption as their motion model. However, trajectories estimated by Kalman filtering often yield suboptimal bounding boxes. In recent approaches, Kalman filtering is employed to estimate the aspect ratio of bounding boxes, which further exacerbates inaccuracies in width estimation. Moreover, since SORT-based algorithms primarily rely on IoU for matching, their performance is highly dependent on the quality of trajectory prediction boxes. Therefore, in complex scenarios (e.g., non-uniform motion or camera movement), the predicted box positions are prone to deviation, affecting the overall performance of the tracker. To address these issues, BoT-SORT [36] proposes three improvements to tracking by detection:

- Firstly, to address the issue where prediction boxes cannot fully enclose pedestrians during real-world prediction, BoT-SORT modifies the state vector and matrix parameters within the Kalman filter. It employs a discrete Kalman filter to model object motion in the image plane, utilizing a constant velocity model. The aspect ratio of the predicted bounding box is directly adjusted to match its width and height, fundamentally enhancing prediction accuracy.
- Secondly, since Kalman filtering relies on a uniform linear motion model, it struggles to handle nonlinear motion disturbances caused by camera movement. To address this, BoT-SORT proposes a Camera Motion Compensation (CMC) scheme that utilizes image registration techniques to eliminate the impact of global motion on target tracking. Without camera parameters, registration between adjacent frames can approximate camera motion. The traditional Global Motion Compensation (GMC) method is employed: feature points are extracted using OpenCV’s feature extraction algorithms, outliers are filtered out via sparse optical flow, and then the affine transformation matrix for background motion is computed and obtained using RANSAC to compute and obtain the affine transformation matrix for background motion. By integrating motion information from preceding and subsequent frames, the predicted position of the pedestrian bounding box is corrected, ensuring the accuracy of tracking matching.
- Finally, BoT-SORT proposes a cosine distance fusion approach for IoU and ReID (re-identification), combining both metrics through a dynamic weighting strategy to enhance the robustness of data association and achieve more stable detection-tracking pairing. By dynamically adjusting the weights of IoU and ReID cosine distance based on detection box confidence, it addresses the occlusion sensitivity issues caused by relying solely on IoU in traditional tracking. Simultaneously, it mitigates false matches resulting from appearance mutations in ReID features.

**Figure 7:**
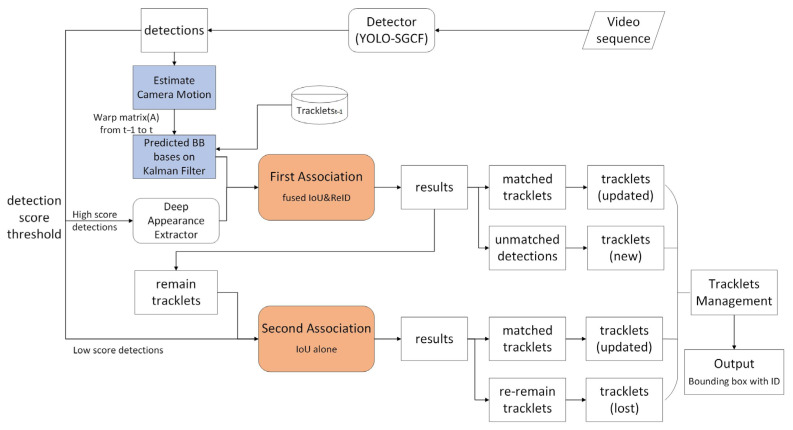
Flowchart of the BoT-SORT-ReID module.

The flowchart of the BoT-SORT-ReID module and its connection to the detection component are shown in Figure 7.

First, the YOLO-SGCF detector processes the video sequence to obtain detections. Next, camera motion is estimated and combined with Kalman Filter predictions for bounding boxes, while depth appearance features are extracted. Then, detection results are categorized by score into high-score detections and low-score detections. High score detections are matched with retained trajectories through a fusion of IoU and ReID first-association matching. Matched trajectories are updated, while unmatched detections generate new trajectories. Low score detections undergo secondary association matching with remaining trajectories post-initial association, based solely on IoU. Matched trajectories are updated, while unmatched trajectories are marked as lost. Finally, trajectory management outputs bounding boxes with IDs.

Through this process, BoT-SORT-ReID can accurately track multiple objects in complex scenes by integrating motion and appearance information, maintaining the continuity of object identities.

## 3. Experimental Results and Analysis

### 3.1. Dataset

**Figure 8:**
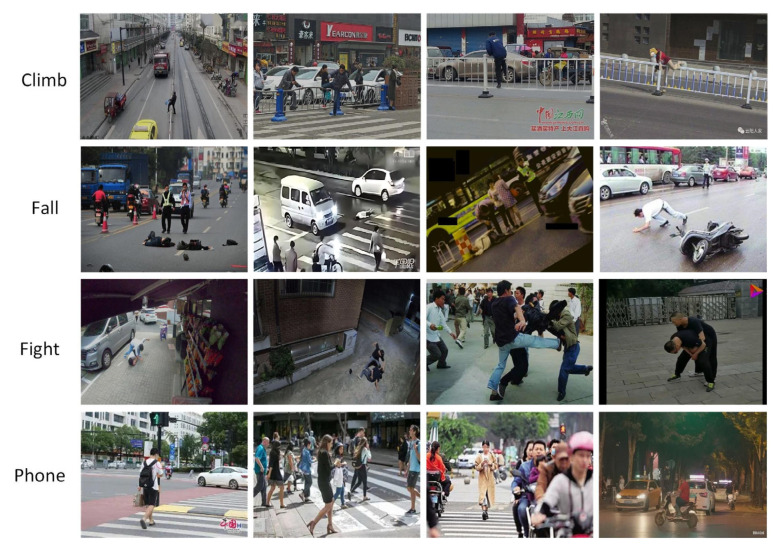
Selected images from different behavioral categories in the dataset.

This paper independently constructed a dataset of pedestrian abnormal behaviors, covering four typical categories: Fall, Fight, Climb, and Phone. The original dataset comprises 6000 images, all uniformly resized to 640 × 640 pixels. These images were collected from diverse urban road scenarios (including intersections, sidewalks, campus roads, etc.), encompassing various lighting conditions such as sunny, cloudy, and nighttime, as well as challenging scenarios involving partial occlusions and complex backgrounds. Due to the difficulty in acquiring Fall and Fight behavior samples, the original dataset suffers from class imbalance. To address this imbalance, a weighted composite augmentation strategy was applied: noise fuzziness, ambient lighting simulation (brightness adjustment ±20%, contrast adjustment ±15%), occlusion enhancement (random rectangular occlusions covering 10–20% area), horizontal flipping, and weighted sampling (assigning 1.5 times sampling weight to Fall and Fight samples). Simultaneously, k-means clustering was employed to redefine the optimal anchor box size tailored to this dataset ([10, 13], [16, 30], [33, 23], [30, 61], [62, 45], [59, 119], [116, 90], [156, 198], [373, 326]). The final dataset was expanded to 11,400 images, randomly split at a 4:1 ratio into a training set (9120 images) and a test set (2280 images). After balancing, the category proportions were Fall:Fight:Climb:Phone = 5:5:4:4. All samples include pixel-level segmentation annotations, with annotation consistency verified to exceed 98.5%, establishing a high-quality data foundation for subsequent model performance evaluation. Figure 8 shows partial image examples from the dataset.

**Figure 9:**
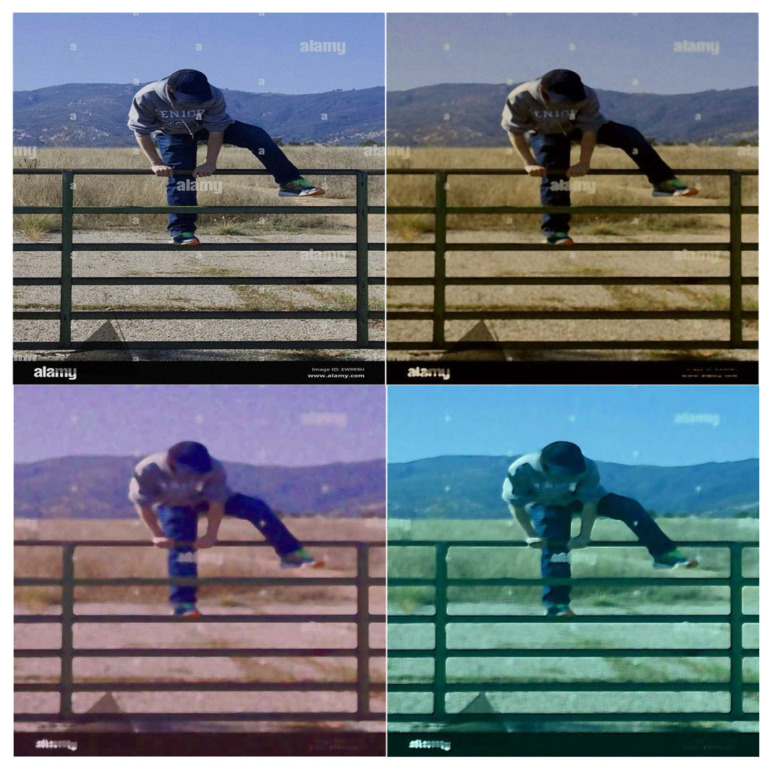
Data augmentation example diagram.

Although noise background replacement may introduce unnatural artifacts, this study cautiously employs this strategy based on the following considerations: (1) In real-world urban surveillance scenarios, pedestrian backgrounds exhibit high uncertainty and diversity. Noise replacement effectively simulates abrupt background changes, reducing model overfitting to specific textures; (2) Compared to alternative approaches: Gaussian blur preserves background semantics but fails to sever feature associations; solid-color masking often causes abrupt foreground-background edge artifacts; GAN-based restoration introduces additional complexity and may generate unreasonable content. Experiments demonstrate that under controlled noise intensity (σ ≤ 30) and usage probability (*p* = 0.2), this strategy enhances the model’s cross-scenario generalization capability by approximately 2.1% without significantly compromising detection accuracy in normal backgrounds (decrease <0.5%). The final enhancement scheme prioritizes physically plausible blurring and occlusion, with noise replacement serving only as a supplementary measure. Therefore, considering computational efficiency, implementation complexity, and the effectiveness of improving model generalization, this study ultimately selected noise background replacement as one of the enhancement strategies. Figure 9 illustrates the same photograph after undergoing different data augmentation techniques.

### 3.2. Experimental Platform and Parameter Settings

The experimental training environment utilizes the Windows 10 operating system, an AMD R7 processor, and an NVIDIA RTX 2060 Ti GPU. Python version 3.8 is employed, with the PyTorch 1.13.0 deep learning framework and CUDA 11.6 parallel computing framework. The development environment is PyCharm 2021. The data augmentation hyperparameters are set as follows: hsv_h: 0.015, hsv_s: 0.7, hsv_v: 0.4, flipud: 0.2, mosaic: 0.8, mixup: 0.15. Other critical training strategy hyperparameters for the model are shown in Table 1.

### 3.3. Evaluation Indicators

#### 3.3.1. Object Detection Evaluation Metrics

To validate the effectiveness of the proposed model, the experiment compares the performance of various models using universally recognized evaluation metrics in the field of object detection. The specific metrics include Precision (P), Recall (R), Average Precision (AP), Mean Average Precision (mAP), Parameters, Giga Floating-point Operations Per Second (GFLOPs), and Frames Per Second (FPS).

Precision represents the proportion of actual positive samples among all results predicted as “positive samples” by the model. Recall denotes the proportion of actual positive targets successfully predicted as positive samples by the model. Their formulas are:

(14) $$P=\frac{TP}{TP+FP}$$

(15) $$R=\frac{TP}{TP+FN}$$

In the formula, T and F represent the actual positive and negative sample classifications, while P and N denote the predicted positive and negative sample classifications. TP stands for true positives, indicating that the target sample was successfully detected—that is, abnormal behavior was correctly identified. FP represents false positives, signifying a misclassification where a negative sample was erroneously predicted as positive—that is, abnormal behavior was identified as non-abnormal behavior. FN denotes a false negative, where a non-target sample is correctly classified as negative, meaning non-abnormal behavior is correctly identified as non-abnormal.

The F1 score is the harmonic mean of precision and recall, serving as a comprehensive measure of a model’s accuracy and recall capability to avoid the limitations of relying on a single metric. Its formula is:

(16) $$F1=\frac{2\times P\times R}{P+R}$$

Plotting the PR curve with R as the horizontal axis and P as the vertical axis visually demonstrates a model’s precision and recall performance across different thresholds. Average Precision (AP) refers to the area under the PR curve. Mean Average Precision (mAP) is the average of AP across all classes, serving as the primary metric for evaluating object detection models. It comprehensively assesses detection performance across all categories, balancing precision and robustness. mAP@0.5 is the mean average precision at an IoU threshold of 0.5. It requires relatively relaxed localization accuracy for predicted boxes and is suitable for rough evaluation. mAP@0.5:0.95 calculates the mean average precision across IoU thresholds from 0.5 to 0.95. It imposes stricter localization accuracy requirements on predicted boxes compared to mAP@0.5, providing a more comprehensive reflection of the model’s localization precision. This metric is particularly suitable for evaluating tasks demanding high localization accuracy. The mAP formula is:

(17) $$mAP=\frac{1}{k}\sum_{i=1}^{k}\int_{0}^{1}P(R)dR$$

where k represents the number of categories.

GFLOPs measures the total number of floating-point operations required during a single forward pass of a model, reflecting its computational complexity and indirectly indicating its operational efficiency on specific hardware devices. A higher GFLOPs value indicates greater computational demands, enabling the model to handle complex, high-precision tasks. Parameters directly reflect the model’s storage space requirements, serving to evaluate resource consumption and deployment feasibility. Therefore, considering both GFLOPs and Parameters as metrics helps evaluate and optimize a model’s performance on specific hardware.

FPS indicates the number of image frames a model can process per second, serving as a measure of the model’s detection rate and a key metric for evaluating its real-time performance. A higher FPS signifies the model’s ability to respond more promptly to dynamic scenes, capture details of fast-moving targets with greater accuracy, and deliver superior real-time processing capabilities.

#### 3.3.2. Tracking Algorithm Evaluation Metrics

The evaluation metrics for the tracking component adopt commonly used indicators for multi-object tracking: MOTA, MOTP, IDS, and IDF1.

IDS (Identity Switch Count) represents the number of times the same target is incorrectly assigned a different identity ID during tracking, directly reflecting the stability of identity recognition. The lower the IDS value, the higher the tracking consistency and accuracy. Multi-Object Tracking Accuracy (MOTA) serves as a comprehensive metric for evaluating a tracking algorithm’s performance in terms of detection errors (misses, false positives) and identity switching. It is one of the most critical indicators in multi-object tracking. The calculation formula is:

(18) $$MOTA=1-\frac{FP+FN+IDS}{GT}$$

where GT denotes the total number of true targets.

Multi-Object Tracking Precision (MOTP) measures the positional matching accuracy between tracking boxes and true object boxes, reflecting the precision of target localization. A higher MOTP value closer to 1 indicates greater overlap between predicted and true object boxes, signifying more accurate target localization. The calculation formula is:

(19) $$MOTP=\frac{\sum_{t,i}d_{t,i}}{\sum_{t,i}1({match}_{t,i})}$$

where $d_{t,i}$ denotes the distance between the bounding box of the i-th matched pair and the ground truth box in frame t (typically the inverse of the IOU or another distance metric); $1({match}_{t,i})$ is an indicator function that equals 1 when a valid match exists for the i-th object in frame t, and 0 otherwise.

IDF1 (Identity F1 Score) is a multi-object tracking F1 score that combines the recall and precision metrics from object detection. It evaluates tracking performance from an identity recognition perspective, where values closer to 1 indicate superior algorithmic performance in both identity recognition and tracking. The calculation formula is:

(20) $$IDF1=\frac{2\times IDTP}{IDTP+IDFP+IDFN}$$

where IDTP represents the number of correct identity matches, i.e., the number of tracked targets whose identities successfully matched their actual identities. IDFP denotes the number of incorrect identity matches, i.e., the number of tracked targets whose identities did not match their actual identities. IDFN indicates the number of unmatched actual targets, i.e., the number of actual targets that were not tracked.

### 3.4. Object Detection Experiments and Results Analysis

#### 3.4.1. Comparative Experiments of Different Loss Functions

To validate the optimization effect of the Focal-EIoU loss function on model detection capabilities, experiments were conducted comparing Focal-EIoU against CIoU (base), EIoU, SIoU, WIoU, and Focal-EIoU. The results are shown in Table 2.

It can be observed that EIoU and Focal-EIoU achieve the best detection performance. Compared to CIoU, Focal-EIoU and EIoU improved the mAP@50 by 1.03% and 1.01%, respectively, effectively enhancing the accuracy of model detection.

Although Focal-EIoU’s single-threshold mAP@50 is slightly lower than EIoU, its Focal mechanism optimizes sample balance, achieving comprehensive improvements in mAP@50:95, precision, and recall across multiple thresholds. This makes it better suited for complex real-world scenarios. Moreover, Focal-EIoU exhibits lower Cls_loss than EIoU, indicating superior object category discrimination and closer alignment between model predictions and ground truth. In contrast, EIoU holds an advantage only in single-threshold accuracy, while its overall performance and adaptability fall short of Focal-EIoU. Therefore, after a comprehensive evaluation, Focal-EIoU is selected as the loss function for this paper.

**Figure 10:**
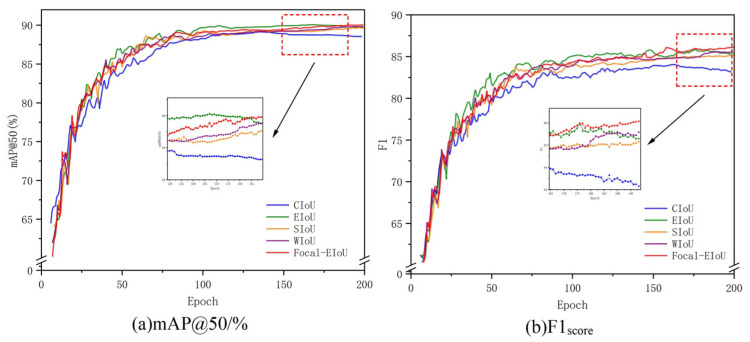
Comparison of loss function training curves and local enlargements. (The arrow points to the enlarged detail, and the dashed box marks the corresponding region in the main figure.) (**a**) shows the training curve for mAP@50/%, while (**b**) displays the training curve for F1 score.

Figure 10 shows the comparative training curves of five loss functions across 1–200 training epochs. The Focal-EIoU curve exhibits a faster rate of ascent, indicating that Focal-EIoU enables the model to converge more rapidly during the initial training phase, accelerating the acquisition of effective parameters. Moreover, its training process exhibits greater stability with reduced fluctuations, meaning Focal-EIoU delivers a more consistent learning journey. This stability helps the model reliably capture target features and patterns while minimizing performance instability caused by excessive training variability.

(To maintain the chart’s visual clarity and aesthetics, data on the *y*-axis has been truncated. The omitted value ranges show minimal variation among the curves.)

#### 3.4.2. Comparative Experiment of Different Attention Mechanisms

To evaluate the effectiveness of the proposed hybrid attention mechanism in enhancing the model’s detection capabilities, experiments were conducted using three single attention mechanisms—CA, CBAM, and SE—along with two attention combinations: SE + CBAM and SE + CA. The performance of the proposed hybrid attention mechanism was compared against these alternatives. The experimental results are shown in Table 3.

From the perspective of core detection metrics, the hybrid attention mechanism combining CA and CBAM demonstrates significant advantages: mAP@50 achieved 90.78%, outperforming single or combined mechanisms such as SE (90.28%) and CA (89.97%). This indicates higher fundamental detection accuracy for targets, enabling more precise identification and localization. Additionally, the CA + CBAM combination achieves a mAP@50:95 of 56.41%, demonstrating robust performance under higher confidence thresholds and adaptability to complex, dynamic pedestrian anomaly detection scenarios. Moreover, CA + CBAM achieved a Precision and Recall of 89.63% and 85.2%, respectively, outperforming other attention mechanisms. High precision implies low false alarm rates and more reliable detection results, while high recall ensures comprehensive capture of abnormal pedestrians, reducing the risk of missed detections. After a comprehensive evaluation of these metrics, CA + CBAM demonstrates a significant overall performance advantage. Therefore, this paper ultimately selects the CA + CBAM hybrid attention mechanism for detection.

**Figure 11:**
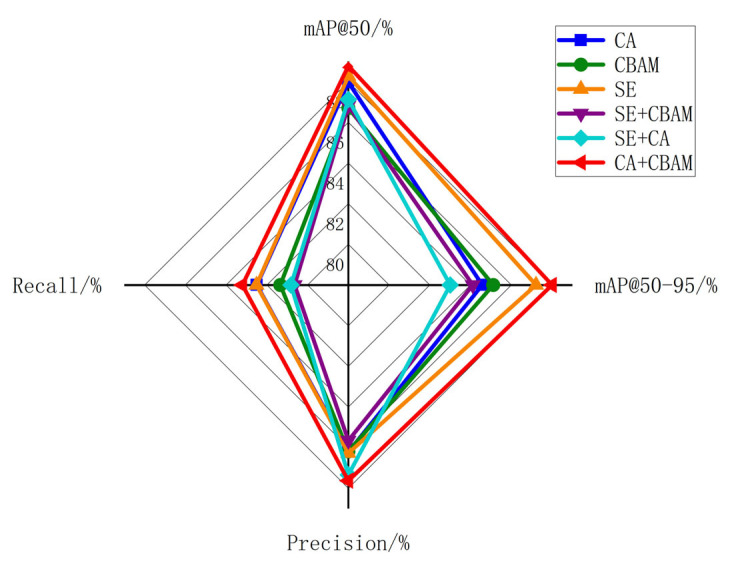
Multi-Indicator Radar Comparison Chart for Different Attention Combinations.

To more intuitively illustrate the comprehensive performance differences among various attention mechanisms, Figure 11 presents a radar chart showing the performance of six mechanisms across four metrics. (Note that the mAP@50–95 data has been linearly scaled and projected to the [85, 90] range to align with the scales of other metrics.) The figure reveals that the polygon outline of the CA + CBAM combined mechanism lies closer to the outer periphery of the radar chart across all metric dimensions. This visually demonstrates its comprehensive advantage across multiple metrics, aligning with the quantitative results presented in Table 3.

**Figure 12:**
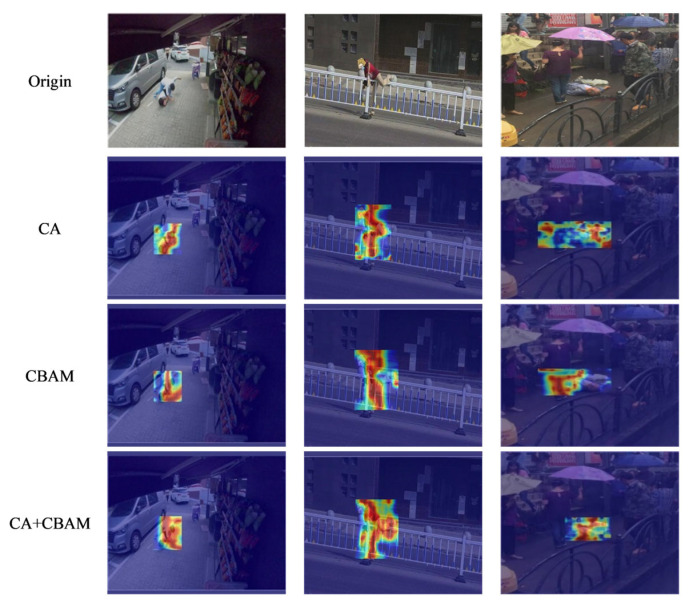
Visualization of CA, CBAM, and CA + CBAMs.

The visualization in Figure 12 further demonstrates that the CA + CBAM attention mechanism better focuses on critical regions. The integration of these two attention types achieves complementary advantages between spatial and channel attention, enabling more comprehensive and detailed extraction of pedestrian features in images. This enhances the model’s ability to express characteristics of abnormal pedestrian behaviors.

#### 3.4.3. Ablation Experiment

To precisely evaluate the specific impact of each improvement component on model performance, YOLOv8n was used as the baseline. Through conducting 11 sets of ablation experiments, the contribution value of each component was analyzed individually. Each experiment strictly controlled consistency in experimental environment configurations and parameters to ensure impartiality of results, as shown in Table 4. In the table, ST, GS, Att, and F-E represent the Swin Transformer module, GhostConv convolution, CA+CBAM hybrid attention, and Focal-EIoU loss function, respectively. Paras denotes Parameters. The symbol “√” indicates that the corresponding module was employed in that experimental group.

As shown in Table 4, Group 1 used no additional modules, employing the original YOLOv8n model as the baseline. Groups 2–5, respectively, incorporated single modules: Swin Transformer, GhostConv, CA + CBAM, and Focal-EIoU. Compared to the original model, mAP@50, mAP@50:95, Precision, and Recall were significantly improved, indicating that these modules better extract features and enhance detection accuracy. Although adding Swin Transformer and attention modules increases parameter count and reduces FPS, the improvement in detection accuracy is substantial. In scenarios demanding high detection precision, this accuracy gain compensates for the speed loss, enabling the model to deliver superior performance in object detection accuracy.

Groups 6 and 7, respectively, incorporate GhostConv convolutions and Focal-EIoU loss functions into the Swin Transformer architecture, achieving improvements in mAP@50, mAP@50:95, and Recall. This demonstrates synergistic effects in feature extraction when combined, balancing local detail capture capabilities. Simultaneously, this approach reduces Parameters and GFLOPs, alleviating the “heavy model” issue inherent to Swin Transformers. Group 8 incorporates GhostConv convolutions into the Focal-EIoU loss function, thereby maintaining the accuracy advantages of Focal-EIoU while achieving comprehensive improvements in mAP@50, mAP@50:95, Precision, and Recall by 1.25%, 5.5%, 1.34%, and 2.58%, respectively, while optimizing feature extraction for small objects. It also reduces computational costs and enhances inference speed. Group 9 incorporates GhostConv convolutions on top of Group 7, achieving positive improvements in mAP@50, Recall, Parameters, and GFLOPs. Although Precision and FPS show slight declines, the overall balance between accuracy and efficiency is superior. Group 10, which incorporates a CA + CBAM hybrid attention mechanism on top of Group 7, only marginally improves partial precision. Its impact on other performance metrics is negligible, necessitating complementary multi-module integration and fusion with other components to fully realize its potential.

Group 11 utilizes all modules, achieving improvements of 3.4%, 8.1%, 4.4%, and 6.0% in mAP@50, mAP@50:95, Precision, and Recall, respectively, compared to the original model, effectively enhancing baseline detection accuracy. However, the increased complexity from stacking multiple modules—particularly the dynamic learning of attention weights—demands additional computational resources, leading to higher Parameters and GFLOPs requirements while sacrificing some inference speed. Overall, the synergistic effect of multiple modules delivers significant improvements across all accuracy metrics, achieving an optimal balance between precision and efficiency. Therefore, this combination is selected.

**Figure 13:**
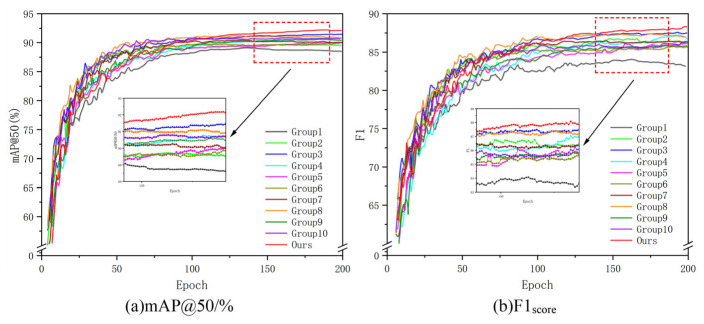
Comparison of Training Curves from Ablation Experiments and Local Magnified Images. The 11 curves correspond to the 11 sets of ablation experiments in Table 4, with the red dashed area enlarged for emphasis.

As shown in the comparative training curve in Figure 13, during the initial training phase, the curve of the proposed algorithm rises significantly faster than the others. This indicates that the model can rapidly learn effective features within a shorter timeframe, thereby enhancing detection performance. Furthermore, it enters the stable phase more quickly—achieving convergence faster—demonstrating that the model completes training more efficiently, reducing training time costs. After convergence, the curve exhibits greater stability, indicating reliable and consistent model performance. This prevents significant metric fluctuations caused by training instability, thereby ensuring the model’s consistency and reliability in practical applications.

#### 3.4.4. Comparative Experiments of Different Algorithms

**Figure 14:**
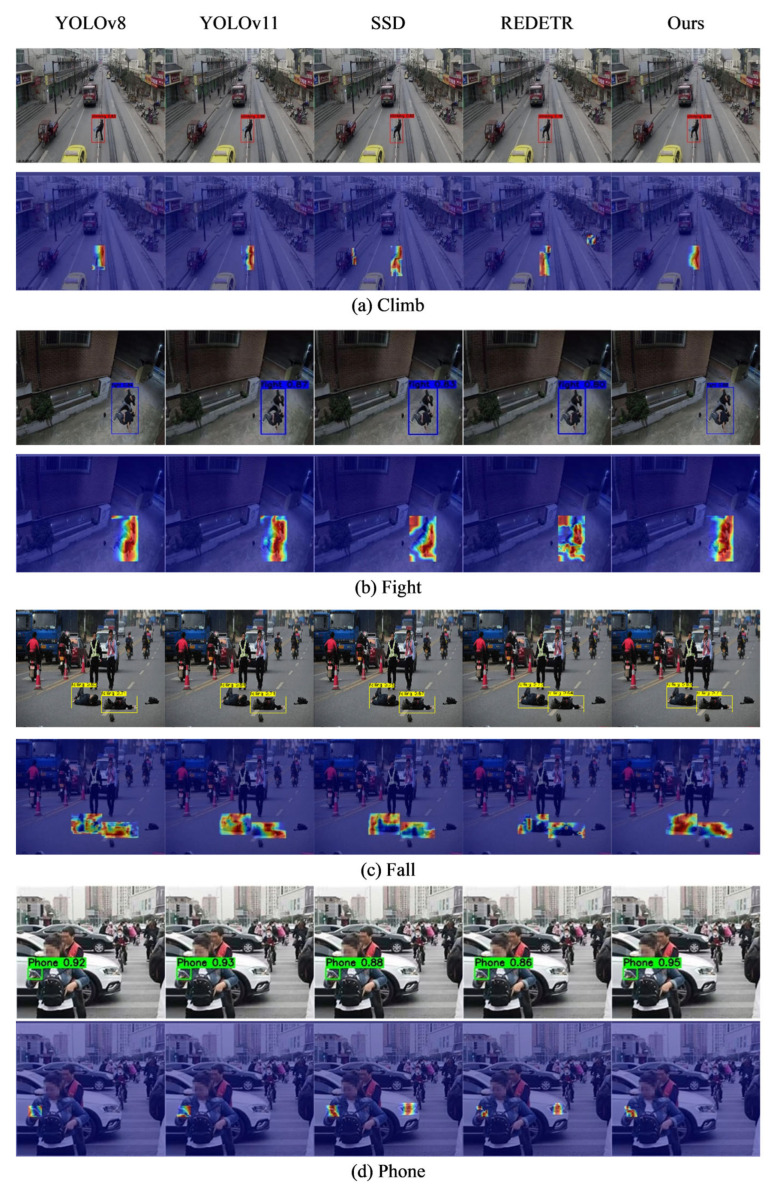
Visualization of algorithm comparison experiments. (**a**) results for “Climb” behavior, with detection regions and confidence scores indicated by red boxes; (**b**) represents “Fight” behavior (blue boxes); (**c**) shows “Fall” behavior (yellow boxes); (**d**) results for “Phone” behavior (green boxes). Each subfigure compares detection outputs of YOLOv8, YOLOv11, SSD, REDETR and the proposed algorithm (Ours).

To fully demonstrate the superiority of the proposed algorithm in object detection tasks, the experiments employ a comparative approach against current mainstream object detection algorithms: widely recognized industry-standard algorithms with extensive real-world applications are selected as benchmarks. Performance evaluations are conducted under identical experimental datasets, hardware environments, and assessment criteria to comprehensively compare the detection capabilities of the proposed algorithm against each mainstream alternative. The experimental results are shown in Table 5, and the visualizations are presented in Figure 14.

As shown in Table 5, the proposed algorithm demonstrates outstanding performance across multiple key metrics. mAP@50 and mAP@50–95 reach 92.2% and 58.22%, respectively, representing improvements of 3.4% and 8.1% over the original algorithm. Precision and Recall also achieve 90.75% and 86.57%, respectively. Although metrics such as Parameters, GFLOPs, and FPS decreased compared to the initial algorithm, the computational complexity was reduced while still meeting the requirements of current real-time tasks. Overall, the performance is well-balanced and surpasses other mainstream object detection algorithms such as YOLOv9, YOLOv11, and SSD.

From the visualization results, compared to algorithms like YOLOv8 and YOLOv11, the Grad-CAM heatmap of this paper’s algorithm (second row in the figure) clearly focuses on the contact areas between the climber’s limbs (hands and legs) and the fence, precisely pinpointing the core actions of abnormal behavior. In Figure 14b Fight, the heatmap of this paper’s algorithm concentrates solely on the interaction zones of the combatants’ limbs, effectively eliminating background interference. The detection bounding boxes also stably frame the combat targets without any missed detections. For the small target image (c) depicting a person falling to the ground, the heatmap precisely focuses on the fallen subject’s torso region. Simultaneously, the detection box fully encloses the fallen target, preventing missed detections. In Figure 14d, the heatmap concentrates on the pedestrian’s hand holding a mobile device, precisely capturing the key action feature of “handheld device.” The detection box’s confidence level also exceeds that of the comparison algorithm, reducing false positives.

#### 3.4.5. Generalization Experiment

Building upon the aforementioned experiments, this paper selects the widely used international public dataset UCSD Ped1 as the validation dataset to verify the generalization effectiveness of the algorithm proposed in this paper across different datasets. The UCSD-ped1 dataset comprises 70 videos of human behaviors captured in outdoor settings. These videos were recorded using a static overhead camera at 10 frames per second, focusing on pedestrians walking. The primary moving objects in these scenes are pedestrians, representing normal behavior. All other objects or abnormal pedestrian movement patterns—such as bicycles, skateboards, wheelchairs, etc.—are classified as abnormal behavior. Due to the presence of a large number of non-abnormal frames in the UCSD Ped1 dataset, pruning was performed to extract all abnormal frames and re-label all abnormal behaviors. The adjusted dataset comprises 4800 images, including 6 types of abnormal behaviors: Biker, Skater, Car, Wheelchair, Lawn (representing the person through the lawn), and Runner. It is split into a training set and a validation set in a 7:3 ratio.

This paper compares the proposed algorithm with commonly used object detection algorithms such as the YOLO series, SSD, Faster R-CNN, REDETR, et.al. The results are shown in Table 6. The table demonstrates that the proposed model achieves a 1.4% improvement in Map over the baseline YOLOv8 model and also outperforms other models. Furthermore, the detection accuracy of the proposed model exceeds 90% for most specific abnormal behavior categories, with particularly significant improvements in the Wheelchair and Lawn categories. This indicates the effectiveness of the proposed model and its strong generalization capabilities.

**Figure 15:**
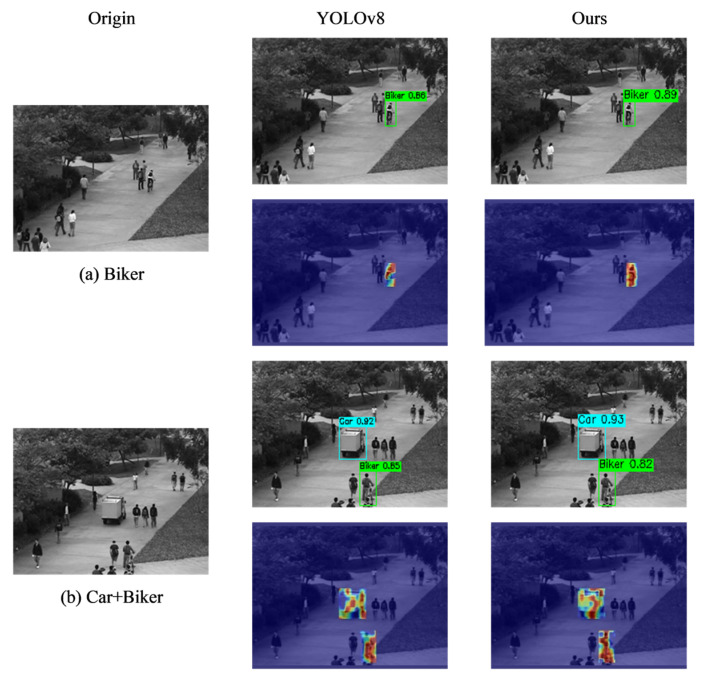
Visual comparison of the YOLOv8 algorithm and the proposed algorithm. (**a**) shows detection results for “Biker,” while (**b**) shows results for “Car + Biker.” Bounding boxes, labels, and confidence scores in the subfigures represent the detection outputs of the algorithms.

Figure 15 presents visualizations for the YOLOv8 algorithm and the proposed algorithm. In image (a), which contains only the Biker category, YOLOv8 successfully detected the target with a confidence score of 0.66. The heatmap shows some response in the Biker region, but the color distribution is relatively scattered, indicating that the model’s focus on the target is moderate. Our model also detected the Biker object with a confidence score of 0.89. The heatmap shows a more concentrated response and more prominent color in the Biker region, indicating that this method achieves better object attention and localization accuracy. Photo (b) contains both the Car and Biker categories. Both models successfully detected both categories, but the proposed model achieved higher confidence scores for both targets than the baseline model. Additionally, the heatmaps show more concentrated responses in the Car and Biker regions, indicating that this method provides more precise attention and localization for each target during multi-object detection.

### 3.5. Object Tracking Experiment Results and Analysis

**Figure 16:**
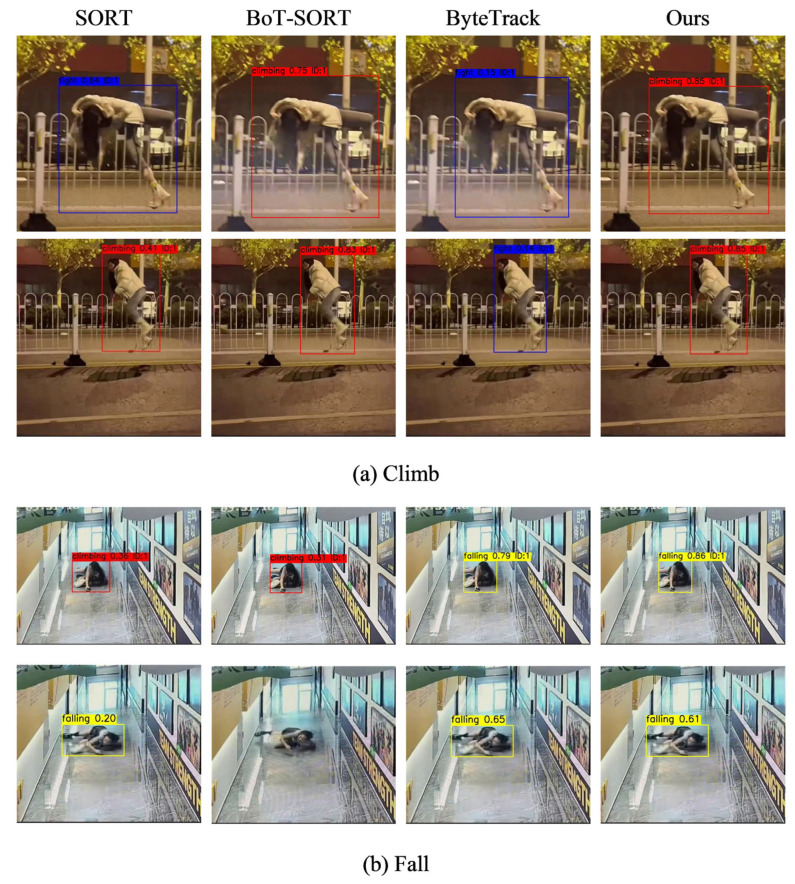
Target tracking visualization diagram. (**a**) shows tracking results for the “Climb” abnormal behavior, while (**b**) presents tracking results for the “Fall” abnormal behavior. The performance of SORT, BoT-SORT, ByteTrack, and the proposed method (Ours) is compared. The bounding boxes and labels in each subfigure indicate the tracked targets and their corresponding behavior categories.

To validate the performance of the proposed algorithm in multi-object pedestrian tracking tasks, we conducted experiments on a self-built dataset and compared it with current mainstream tracking algorithms (SORT, BoT-SORT, and ByteTrack). The experiments comprehensively evaluated the performance of each algorithm using multiple metrics, including MOTA, MOTP, IDS, IDF1, and FPS. The results are shown in Table 7, and the target tracking visualization is depicted in Figure 16.

Experimental results demonstrate that the proposed algorithm achieves optimal performance across multiple evaluation metrics. Specifically, ReID-Bot achieves a MOTA of 90.8% and a MOTP of 92.6%, significantly outperforming the comparison algorithms. Additionally, the number of false detections (IDS) was reduced to 11, while the IDF1 reached 80.9%, demonstrating the algorithm’s outstanding capability in maintaining pedestrian identity and effectively reducing loss of tracking and ID confusion. Although slightly slower than algorithms like Bytetrack, it still maintains near real-time performance at approximately 20 FPS, making it practical for real-world applications.

The object tracking visualization diagram provides an intuitive observation of each algorithm’s tracking performance under Climb and Fall anomaly behaviors. Both the SORT and ByteTrack algorithms exhibit varying degrees of misclassification. For instance, the SORT algorithm misclassified Climb (red box) as Fight (blue box) and Fall (yellow box) categories. Meanwhile, the BoT-SORT algorithm also exhibits false negatives, as evidenced by the Fall target in Figure 16b that remains untracked. In contrast, our algorithm consistently encloses pedestrian targets with tighter, more precise tracking boxes across all scenarios. It exhibits virtually no false detections (tracking boxes accurately correspond to targets without including extraneous background or misidentifying other objects) or false negatives (targets remain entirely within tracking boxes without loss). This further validates the model’s tracking continuity and accuracy in complex environments, confirming the performance advantages reflected in the metrics presented in Table 7.

## 4. Conclusions

To address the challenges faced by existing pedestrian anomaly detection methods in complex traffic scenarios—such as poor real-time performance, low accuracy, and high false-negative rates—this paper proposes a multi-module collaborative fusion detection and tracking framework based on an improved YOLOv8 detector and a BoT-SORT-ReID tracking module. The detection module incorporates a Swin Transformer to enhance global feature modeling capabilities and integrates a hybrid attention mechanism combining CA and CBAM to improve focus on key regions and channel features. GSConv convolutions optimize feature fusion while reducing computational load, and Focal-EIoU loss addresses bounding box regression and sample imbalance. The tracking module integrates motion compensation with ReID-feature-based association strategies, significantly improving identity consistency and long-term tracking stability.

Experiments on a self-built dataset demonstrate that the proposed detection model YOLO-SGCF achieves mAP@50, mAP@50:95, Precision, and Recall on our self-built dataset reached 92.2%, 58.22%, 90.75%, and 86.57%, respectively. Compared to the original YOLOv8n model, these metrics improved by 3.4%, 8.1%, 4.4%, and 6.0%, respectively. The model also outperformed current mainstream algorithms in recognition performance. For multi-object tracking tasks, the proposed method achieved 90.8% MOTA and 92.6% MOTP, with identity switch count (IDS) reduced to 11. This significantly outperforms mainstream tracking algorithms like SORT, BoT-SORT, and ByteTrack, while maintaining near-real-time processing speed (approximately 20 FPS) and effectively enhancing tracking continuity in complex scenes.

Furthermore, to validate the model’s generalization capability, cross-scenario testing on the public UCSD Ped1 dataset yielded a mAP@50 of 92.7%, outperforming comparison models like YOLOv8, YOLOv9, SSD, and Faster R-CNN. This further demonstrates its adaptability and stability across diverse road environments.

Although the proposed method demonstrates strong detection and tracking performance, several limitations remain: First, model stability under extreme occlusions, severe lighting variations, or dense crowd scenes still has room for improvement. Second, the relatively high number of model parameters and computational complexity pose challenges for real-time deployment on resource-constrained edge devices.

Future work will focus on the following directions: First, exploring lightweight techniques such as knowledge distillation and network pruning to further enhance inference efficiency while maintaining accuracy. Second, constructing richer, multi-scenario pedestrian abnormal behavior datasets to improve the model’s generalization and robustness in real-world open environments. Third, researching multimodal fusion mechanisms to combine infrared, depth, and other information to enhance the system’s perception capabilities under all-weather conditions.

## Figures and Tables

**Table 1:** Key parameter settings for Model Training.

Parameter	Parameter Value
imgsz	640 × 640
epoch	200
batch_size	16
lr0	0.001
lrf	0.005
momentum	0.937
weight_decay	0.001
dropout	0.15
mask_ratio	4
optimizer	AdamW

**Table 2:** Comparative experiments of different loss functions.

Loss	mAP@50/%	mAP@50–95/%	Precision/%	Recall/%
CIoU	89.14	53.85	86.95	81.69
EIoU	90.06	54.84	89.34	83.24
SIoU	89.70	54.51	87.30	83.91
WIoU	89.81	54.92	88.51	83.05
Focal-EIoU	90.04	55.11	89.47	84.02

**Table 3:** Comparative experiment of different attention mechanisms.

Attention	mAP@50/%	mAP@50–95/%	Precision/%	Recall/%	F1
CA	89.97	54.46	88.21	84.49	86.31
CBAM	88.70	54.77	88.19	83.34	85.70
SE	90.28	55.96	88.27	84.51	86.35
SE + CBAM	88.82	54.20	87.65	82.64	85.07
SE + CA	89.12	53.57	89.34	82.82	85.96
CA + CBAM	90.78	56.41	89.63	85.20	87.36

**Table 4:** Comparison of Ablation Experiment Results.

Group	ST	GS	Att	F-E	mAP		Precision	Recall	Paras/10^6^	GFLOPs	FPS
					@50/%	@50:95/%					
1					89.14	53.85	86.95	81.69	3.01	8.1	348.06
2	√				89.70	55.75	90.14	84.06	2.85	10.4	324.49
3		√			91.50	58.83	90.56	85.83	2.90	7.7	362.74
4			√		90.78	56.41	89.63	85.2	4.29	13.6	294.05
5				√	90.04	55.11	89.47	84.02	2.99	7.8	358.91
6	√	√			89.95	56.88	89.93	84.12	2.76	7.6	333.37
7	√			√	90.43	56.44	89.90	84.88	2.85	7.7	336.54
8		√		√	91.17	58.14	90.67	86.19	2.70	7.7	363.96
9	√	√		√	90.81	55.67	88.42	85.22	2.76	7.6	311.36
10	√		√	√	90.60	55.50	88.43	83.93	4.15	12.5	307.51
11	√	√	√	√	92.20	58.22	90.75	86.57	3.26	9.4	328.49

**Table 5:** Experimental Results Comparing the Performance of Different Algorithms (Mean ± std, *n* = 5).

Model	mAP		Precision	Recall	Paras/10^6^	GFLOPs	FPS
	mAP@50/%	mAP@50:95/%					
YOLOv8n	89.14 ± 0.21	53.85 ± 0.38	86.95 ± 0.26	81.69 ± 0.32	3.01	8.1	348.06
YOLOv5	84.06 ± 0.24	56.09 ± 0.41	80.13 ± 0.31	78.95 ± 0.36	2.50	7.1	373.33
YOLOv9	90.47 ± 0.19	57.46 ± 0.35	89.74 ± 0.23	84.43 ± 0.3	7.17	26.7	281.16
YOLOv11	91.41 ± 0.18	58.75 ± 0.33	89.62 ± 0.22	85.24 ± 0.28	2.58	6.3	395.27
SSD	88.83 ± 0.24	53.41 ± 0.42	89.29 ± 0.28	81.78 ± 0.34	—	—	—
Faster R-CNN	82.82 ± 0.27	52.50 ± 0.45	82.41 ± 0.33	79.11 ± 0.38	—	—	—
REDETR	83.04 ± 0.26	51.32 ± 0.46	84.46 ± 0.3	79.03 ± 0.37	31.99	103.4	157.36
FCOS	87.64 ± 0.23	53.71 ± 0.39	84.97 ± 0.29	80.58 ± 0.35	36.3	86.1	198.25
Ours	92.20 ± 0.16	58.22 ± 0.32	90.75 ± 0.21	86.57 ± 0.27	3.26	9.4	328.49

All metrics represent the mean ± standard deviation from five independent replicate experiments. Our proposed algorithm significantly outperformed all comparison models on mAP@50 (paired *t*-test, *p* < 0.001, Bonferroni corrected) and achieved the highest recall (*p* < 0.01). Optimal values are bolded; “—” indicates unavailable data.

**Table 6:** Comparison of Generalization Performance Across Different Algorithms on the UCSD-Ped1 Dataset (Mean ± std, *n* = 5).

Method	AP/%						mAP/%	Paras/10^6^	GFLOPs	FPS
	Biker	Skater	Car	Wheelchair	Lawn	Runner				
SSD	80.4 ± 1.8	79.8 ± 1.9	88.9 ± 1.5	89.9 ± 1.4	70.2 ± 2.5	74.1 ± 2.3	80.55 ± 1.7	—	—	—
Faster R-CNN	79.5 ± 1.9	77.1 ± 2.1	90.1 ± 1.4	88.5 ± 1.5	71.4 ± 2.4	72.6 ± 2.4	79.9 ± 1.8	—	—	—
YOLOv5	97.3 ± 0.4	97.5 ± 0.4	98.2 ± 0.3	93.6 ± 0.7	79.8 ± 1.8	73.6 ± 2.4	90 ± 1.1	2.5	7.1	373.33
YOLOv8	98.9 ± 0.3	98.7 ± 0.3	98.5 ± 0.3	93 ± 0.8	81.3 ± 1.7	77.4 ± 2.2	91.3 ± 0.9	3.01	8.1	348.06
YOLOv9	98.3 ± 0.4	98.6 ± 0.3	98.7 ± 0.3	92.7 ± 0.8	83.3 ± 1.6	79.2 ± 2.1	91.8 ± 0.8	7.17	26.7	281.16
REDETR	87.9 ± 1.2	85.7 ± 1.4	95.5 ± 0.9	92.4 ± 0.9	77.8 ± 1.9	75.7 ± 2.2	85.8 ± 1.3	31.99	103.4	157.36
FCOS	98 ± 0.4	96.4 ± 0.6	98.1 ± 0.4	92.5 ± 0.8	69.9 ± 2.6	71.9 ± 2.5	87.8 ± 1.4	36.3	86.1	198.25
[37]	87.8 ± 1.3	75.8 ± 2.3	99.5 ± 0.2	95.6 ± 0.7	98 ± 0.4	—	92.7 ± 0.6	8.74	22.3	102
Ours	97.9 ± 0.4	99.1 ± 0.2	98.9 ± 0.3	94.3 ± 0.6	87.7 ± 1.5	78.3 ± 2.2	92.7 ± 0.5	4.08	17.7	328.49

**Table 7:** Pedestrian target tracking comparison results.

Method	MOTA	MOTP	IDS	IDF1	FPS
SORT	88.5	90.1	21	76.7	19.92
BoT-SORT	89.6	91.8	13	79.6	20.2
ByteTrack	89.3	92.1	15	79.2	22.43
Ours	90.8	92.6	11	80.9	19.98

## Data Availability

The original contributions presented in this study are included in the article. Further inquiries can be directed to the corresponding author.
